# Identity development in adolescents with mental problems

**DOI:** 10.1186/1753-2000-7-26

**Published:** 2013-07-31

**Authors:** Emanuel Jung, Oliver Pick, Susanne Schlüter-Müller, Klaus Schmeck, Kirstin Goth

**Affiliations:** 1 Child and Adolescent Psychiatric Hospital, Psychiatric University Hospitals, Basel, Switzerland; 2 Practice for Child and Adolescent Psychiatry, Frankfurt, Germany; 3 University of Applied Sciences FHNW, Basel, Switzerland

**Keywords:** Identity, Assessment, Personality disorder, Adolescence, Psychopathology

## Abstract

**Background:**

In the revision of the Diagnostic and Statistical Manual (*DSM-5*), “Identity” is an essential diagnostic criterion for personality disorders (self-related personality functioning) in the alternative approach to the diagnosis of personality disorders in Section III of DSM-5. Integrating a broad range of established identity concepts, *AIDA* (Assessment of Identity Development in Adolescence) is a new questionnaire to assess pathology-related identity development in healthy and disturbed adolescents aged 12 to 18 years. Aim of the present study is to investigate differences in identity development between adolescents with different psychiatric diagnoses.

**Methods:**

Participants were 86 adolescent psychiatric in- and outpatients aged 12 to 18 years. The test set includes the questionnaire *AIDA* and two semi-structured psychiatric interviews (*SCID-II, K-DIPS*). The patients were assigned to three diagnostic groups (personality disorders, internalizing disorders, externalizing disorders). Differences were analyzed by multivariate analysis of variance MANOVA.

**Results:**

In line with our hypotheses, patients with personality disorders showed the highest scores in all AIDA scales with T>70. Patients with externalizing disorders showed scores in an average range compared to population norms, while patients with internalizing disorders lay in between with scores around T=60. The AIDA total score was highly significant between the groups with a remarkable effect size of f= 0.44.

**Conclusion:**

Impairment of identity development differs between adolescent patients with different forms of mental disorders. The *AIDA* questionnaire is able to discriminate between these groups. This may help to improve assessment and treatment of adolescents with severe psychiatric problems.

## Background

Identity is a broadly discussed construct and is linked to different psychodynamic [1,2], social cognitive [3,4], and philosophical theories (see Sollberger in this issue). Erikson [1] defines identity as a hybrid concept providing a sense of continuity and a frame to differentiate between self and others, which enables a person to function autonomously. Ermann [5] describes identity similarly as aligned in a transitional space between a given person and his or her community. On the one hand, a person has a sense of uniqueness regarding the past and the future; on the other hand, he or she sees differences as well as resemblances to others. “This sense of coherence and continuity in the context of social relatedness shapes life” [5], p. 139.

Establishing a stable identity is one major development task in adolescence [6]. These challenges of identity formation go along with identity crises that are normal and temporary phenomena in mastering age-related developmental tasks in adolescence [6]. According to Kernberg [7], the transformation of the physical and psychological experiences of young people and the discrepancy between the sense of self and the others’ view of the adolescent lead to identity crises. Erikson [1] emphasizes the need for resolution of identity crises by synthesizing previous identifications and introjections into a consolidated identity.

In contrast to the non-pathological identity crisis, we use the concept of identity diffusion as a pathological identity development that is viewed as a psychiatric syndrome underlying all severe personality disorders [7,8]. According to Kernberg’s theory of personality disorders [9], borderline personality organization is hallmarked by identity diffusion. Patients with identity diffusion have a non-integrated concept of the self and significant others so that a clinician cannot get a clear picture of the patient’s description of himself and of significant others in his life [10]. There is often no commitment to jobs, goals and relationships as well as an avoidance of ambivalence associated with a painful sense of incoherence [11].

Probably due to present changes in society with transitions in family and work, the number of patients with identity diffusion increases over time [5,12,13]. In contrast to the understanding outlined above, other authors (e.g. Marcia’s identity status paradigm [14]) view identity diffusion as a concept containing a broad range from adaptability to psychopathology like borderline personality disorders. From an optimistic point of view, identity diffused individuals are flexible (due to the lack of commitment) and seem to accommodate well to the fast-moving technological world [14]. For other authors [15], post-modern life as a whole is hallmarked by a condition of diffusion. Whether one agrees with the post-modern view or not, the development of healthy and disturbed identity is a topic of high interest. In the following, new conceptualizations, methods of treatment, and diagnostic instruments of healthy and disturbed identity are discussed. Goth et al. [16] presented an integrative understanding of healthy and disturbed identity and developed the self-report instrument AIDA (Assessment of Identity Development in Adolescence) to assess pathology-related identity development in adolescence. In the present study, the potential of *AIDA* is proved by investigating differences in identity development between adolescents with different psychiatric diagnoses.

### New conceptualizations: identity concepts in DSM-5

The *DSM-IV* includes identity disturbance as a criterion of borderline personality disorder and defines it as “markedly and persistently unstable self-image or sense of self” [17], p. 654. In the revision from *DSM-IV* to *DSM-5*[18,19], the concept of identity is a central part of a new conceptualization of personality disorders in the alternative approach to the diagnosis of personality disorders in Section III of DSM-5 (see Schmeck et al. in this issue). The core criteria of personality disorders are composed of impairments in personality functioning in the two domains of self-functioning (self-direction and identity) and interpersonal functioning (empathy and intimacy). Identity is defined as the “experience of oneself as unique, with clear boundaries between self and others; stability of self-esteem and accuracy of self-appraisal; capacity for, and ability to regulate, a range of emotional experience” [20]. The new model is placed in Section III of DSM-5 to stimulate further research in this field.

### New method of treatment: Adolescent Identity Treatment (AIT)

Research of the last 15 years reveals increasing evidence that personality disorders are a prominent form of psychopathology in adolescence [21-24]. Personality disorders prior to age of 18 years can be reliably diagnosed [25,26]. They have a good concurrent [24,27] and predictive validity [22] with adequate internal consistency [28] and similar stability to personality disorders in adulthood [27,29,30]. Thus, symptoms of personality disorders in adolescence can be diagnosed and targeted for treatment [11,31,32]. Paulina Kernberg [10] described a model for understanding the impact of identity diffusion as a pathogenic mechanism in developing a personality disorder in adolescence and stressed the need to differentiate between normal identity crisis and pathological identity diffusion for a targeted therapeutic intervention. These ideas lead to the development of the psychodynamic treatment approach “Adolescent Identity Treatment” (*AIT*) [33]. This treatment focuses on identity diffusion in adolescence and is designed to help young patients to establish satisfying relationships, gain self-esteem and clarify aims in life.

### New diagnostic instrument: the questionnaire AIDA (Assessment of Identity Development in Adolescence)

Our research group developed the questionnaire *AIDA - Assessment of Identity Development in Adolescence*[16] to assess pathology-related identity development in healthy and disturbed adolescents aged 12 to 18 years in self-report for diagnostic and prognostic issues. Thus, *AIDA* is predestinated to be used as a research tool to evaluate therapy efficacy of *AIT* as well as of every therapy addressing improvement in self-related personality functioning related to constructs described below.

Discourses about identity are heterogeneous [12]. With respect to a broad range of theoretical descriptions about identity development, two domains have been distinguished for constructing the *AIDA*. In line with the constructs’ dichotomy in social-cognitive psychology as well as in the psychopathology-oriented psychodynamic descriptions the AIDA model distinguishes between the two dimensions “Continuity” and “Coherence”, serving as a well elaborated theoretical framework to find a meaningful and distinct substructure of the higher order construct “identity integration vs. identity diffusion” (for a detailed description see [16]). Following strict rules of deductive test construction and focusing on clear-cut constructs, we integrated aspects of operationalizations of identity diffusion by other authors like Kernberg [34], Westen [35] and Akhtar & Samuel [36] and additionally differentiated the aspects of psychosocial functioning “self-related“, “social-related“, and “related to mental representations / ability” following e.g. Fonagy (emotional and cognitive self-reflection is viewed as an elementary basis for identity development [37]) in order to substructure the construct along its hypothesized constituents (see Table 1).

**Table 1:** Theory-based suggestion for a meaningful substructure of the construct “Identity Integration vs. Identity Diffusion” and its operationalization into AIDA scales, subscales, and facets

**Identity integration vs. Identity diffusion**		
Scale 1:	Scale 2:	Psychosocial functioning
Identity-Continuity vs. **Discontinuity**	Identity-Coherence vs. **Incoherence**	
Ego-Stability, intuitive-emotional “I” (“Changing while staying the same”)	Ego-Strength, defined “ME” (“non-fragmented self with clear boundaries”)	
Sub 1.1: **Stability in attributes** / goals vs. lack of perspective	Sub 2.1: **Consistent self** image vs. contradictions	**Self-related** intrapersonal “Me and I”
F1: capacity to invest / stabilizing commitment to interests, talents, perspectives, life goals	F1: same attributes and behaviors with different friends or situations, consistent appearance	
F2: stable inner time-line, historical-biographical self, subjective self-sameness, sense of continuity	F2: no extreme subjective contradictions / diversity of self-pictures, coherent self-concept	
F3: stabilizing moral guidelines and inner rules	F3: awareness of a defined core and inner substance	
Sub 1.2: **Stability in relations** / roles vs. lack of affilitation	Sub 2.2: **Autonomy** / ego-strength vs. over-identification, suggestibility	**Social-related** interpersonal “Me and You”
F1: capacity to invest / stabilizing commitment to lasting relationships	F1: assertiveness, ego-strength, no over-identification or over-matching	
F2: positive identification with stabilizing roles (ethnic - cultural - family self)	F2: independent intrinsic self-worth, no suggestibility	
F3: positive body-self	F3: autonomous self (affect) regulation	
Sub 1.3: Positive **emotional self reflection** vs. distrust in stability of emotions	Sub 2.3: positive **cognitve self reflection** vs. superficial, diffuse representations	**Mental representations** accessability and complexity concerning own and others’ emotions / motives
F1: understanding own feelings,good emotional accessibility	F1: understanding motives and behavior, good cognitive accessibility	
F2: understanding others´ feelings, trust in stability of others’ feelings	F2: differentiated and coherent mental representations	

The construct “Continuity” represents the vital experience of “I” and subjective emotional self-sameness with an inner stable time line. High “Continuity” is associated with the stability of identity-giving goals, talents, commitments, roles, and relationships, and a good and stable access to emotions as well as the trust in the stability of them. A lack of Continuity (i.e. high “Discontinuity”) is associated with a missing self-related perspective, no feeling of belonging and affiliation, and a lack of access to emotional levels of reality and trust in the durability of positive emotions.

The construct “Coherence” stands for clarity of self-definition as a result of self-reflective awareness and elaboration of the “ME”, accompanied by consistency in self-images, autonomy and Ego-strength, and differentiated mental representations. A lack of Coherence (i.e. high “Incoherence”) is associated with being contradictory or ambivalent, suggestible and over-matching, and having poor access to cognitions and motives, accompanied by superficial and diffuse mental representations. The scales are coded towards psychopathology. High scores in the AIDA scales “Discontinuity” and “Incoherence” are indicators of an identity diffusion.

The current study contrasts the identity development of personality disordered adolescents with the identity development of adolescents suffering from internalizing or externalizing disorders. In child and adolescent psychiatric research a procedure like this is often used to clarify the question if discrepancies from a normal sample are specific for a special diagnostic group or if they are a characteristic of mental disorders in general. As outlined above, identity problems are one of the core criteria of personality disorders so that we hypothesize adolescents with personality disorders reaching significantly higher scores in identity diffusion in comparison to other clinical groups. Up to now there are no studies about systematic differences in the level of identity problems in non-PD adolescent patients so that our second hypothesis is based on clinical experience. Patients with severe anxiety disorders and major depression experience a substantially reduced self-esteem which could have an impact on identity development. In contrast, patients with externalizing disorders boost their self-esteem by externalizing their problems. Based on these observations we hypothesize elevated scores of identity diffusion in patients with internalizing disorders in comparison with patients with externalizing disorders.

## Methods

### Participants and procedures

Participants were 86 inpatients and outpatients of a child and adolescent psychiatric university hospital (N= 75) and a child and adolescent psychiatric practice (N=11). Inclusion criteria were age 12–18 years, sufficient linguistic and cognitive skills to master the written task and no current psychotic episode. The sample consisted of 30 boys (34.9%) and 56 girls (65.1%) in the age range from 12–18 years (mean age 15.24, SD 1.77). The study was approved by the local ethics committee and written informed consent was given. Taking into account the results of the diagnostic interviews *K-DIPS* (Children – Diagnostic Interview for Psychiatric Diseases) [38] and *SCID-II* (The Structured Clinical Interview for DSM-IV, Axis II) [39] (see below) and of a classification conference, the patients were assigned to one of the three diagnostic groups “personality disorder (PD)”, “internalizing disorder (internal)”, or “externalizing disorder (external)” (see Table 2). Patients who clearly fulfilled the DSM-IV criteria of a personality disorder were allocated to the PD-group independently of axis I comorbidities like anxiety or depression. Patients with internal or external problems were attributed to the correspondent groups, if the diagnoses were unambiguous and no comorbidities were detected. We excluded patients from further analysis if they showed comorbid internalizing and externalizing problems or other psychiatric disorders like psychoses or pervasive developmental disorders.

**Table 2:** Mean score (M) and standard deviation (SD) differences with associated significance level p and effect size f in the different diagnostic groups: personality disorder (PD), internalizing disorder (internal), and externalizing disorder (external)

	**Differences between diagnostic groups**					
	**PD**	**Internal**	**External**			
	**N= 24**	**N= 24**	**N=10**			
	**M (SD)**	**M (SD)**	**M (SD)**	**F**	p*^1^	f*^2^
AIDA total score: **Identity diffusion**	135.96 (27.41)	96.82 (39.22)	60.50 (30.18)	13.485	.000***	0.44
**1. Discontinuity**	58.29 (13.02)	42.23 (18.80)	28.70 (12.66)	9.588	.000***	0.36
1.1 attributes	23.92 (16.05)	19.09 (11.48)	14.40 (6.10)	1.484	.230	0.08
1.2 relationships	20.17 (6.45)	13.00 (7.92)	9.20 (7.38)	7.030	.000***	0.29
1.3 emotional self-refl.	16.29 (5.54)	13.18 (6.65)	5.10 (3.64)	9.751	.000***	0.36
**2. Incoherence**	74.96 (19.21)	51.55 (25.78)	31.80 (22.07)	9.615	.000***	0.36
2.1 consistent self	32.00 (6.24)	20.82 (9.84)	13.50 (9.93)	13.106	.000***	0.43
2.2 autonomy	26.17 (8.60)	19.77 (8.49)	10.20 (8.43)	8.375	.000***	0.33
2.3 cognitive self-refl.	19.50 (5.88)	14.00 (5.97)	8.10 (6.26)	7.279	.000***	0.35

*^1^: Significance p ***=0.1% level, *^2^: effect size f>0.10 small, f>0.25 medium, f>0.40 big.

From the 86 patients,

● N= 24 were assigned to the “PD”-group according to the results of the SCID-II interview (15 Borderline PD (F60.3), 5 other cluster-B PD, 3 cluster-C PD and 1 cluster-A PD).

● N= 22 were assigned to the group “internal” (15 depressive disorders (F33), 5 anxiety disorders (F40) and 2 emotional disorders (F93)).

● N= 10 patients were assigned to the “external”-group (7 ADHD (F90, F90.1, F98.8) and 3 conduct disorder (F91)).

● N= 30 could not be assigned to one of the research groups because of comorbidities or non-target diagnoses.

In this process we took especially care to create “pure” diagnostic groups to enable valid interpretations of differences between these types of psychiatric disorders in terms of differences in identity development.

### Measures

#### AIDA

*AIDA* (Assessment of Identity Development in Adolescence) [40] is a self-report questionnaire for adolescents from 12 to 18 years to assess pathology-related identity development. Its construction was based on a broad description of the field integrating classical approaches and constructs from psychodynamic and social-cognitive theories, focusing on a comprehensive and methodological optimized assessment. The 58 5-step format items were coded towards pathology and add up to a total score ranging from “identity integration to identity diffusion”. To facilitate scientific communication on the one hand and research concerning possible specific relations to external variables on the other hand, the integrated subconstructs constituting “Identity Diffusion” together are formulated in terms of distinct scales and subscales. The differentiated scales and subscales are referring to distinct psychosocial or functional constituents without regarding them to be statistically independent variables (see Table 1).

In a mixed school (N = 305) and clinical sample (N = 52) *AIDA* showed excellent total score (Diffusion: α = .94), scale (Discontinuity: α = .86; Incoherence: α = .92) and subscale (α = .73-.86) reliabilities [16]. Construct validity could be shown by high intercorrelations between the scales supporting as well the subdifferentiation as the subsumed total score. EFA on item level confirmed a joint higher order factor explaining already 24.3% of variance. High levels of Discontinuity and Incoherence were associated with low levels in Self Directedness (*JTCI 12–18 R*[41,42]), an indicator of maladaptive personality functioning. Criterion validity could be demonstrated with both AIDA scales differentiating between patients with a personality disorder (N = 20) and controls with remarkable effect sizes (d) of 2.17 and 1.94 standard deviations. Several translations of *AIDA* in different languages are in progress and show similar promising results concerning psychometric properties (for the Mexican version of *AIDA* see Kassin & Goth, this issue).

#### SCID-II and K-DIPS

As the aim was to explore the thresholds between healthy development, identity crisis and identity diffusion, valid and broad measures for psychopathology were needed. We used the two well-established semi-structured diagnostic interviews *SCID-II*[39] and *K-DIPS*[38]. *SCID-II* (The Structured Clinical Interview for *DSM-IV* Axis II) is designed to assess personality disorders according to *DSM-IV* criteria. Administration time is about 60–90 minutes. *K-DIPS* (Children – Diagnostic Interview for Psychiatric Diseases) is designed to assess axis I psychopathology in children and adolescents according to *ICD-10* and *DSM-IV* criteria, and takes about 90–120 minutes to administer.

### Statistical analysis

We used the Statistical Package for the Social Sciences (SPSS 19 for Windows) for data analyses. Differences between the three groups of psychiatric disorders in AIDA scores were analyzed by multivariate analysis of variance MANOVA with the factor “pathology” (PD, internal, external). The factor “sex” was integrated as a covariate since systematic differences had been detected between boys and girls in the validation sample and different population norms had been suggested [16]. Effect size *f* is supposed to be big with >.40 but should be at least medium with >.25 to avoid overinterpretation of significant group differences. The sample size is sufficient to test for big effect sizes with significance level p<.05.

## Results

In line with our hypotheses, the patients with personality disorders showed the highest scores in all AIDA scales, the patients with externalizing disorders the lowest scores, while the patients with internalizing disorders scored in between (see Table 2). For the AIDA total score “Identity Diffusion” the effect size of this highly significant group difference was big with f= 0.44. The two primary scales “Discontinuity” and “Incoherence” seemed to differentiate with a similar quality between the groups, both reaching nearly big effect sizes with f= 0.36. On AIDA subscale level, distinct potential to differentiate between types of pathology was detected. While the identity component “Incoherence concerning consistent self-picture” differentiated with a big effect size of f= 0.43 between the groups, the subscale “Discontinuity concerning attributes and goals” did not significantly differentiate between the groups. The other subscales all reached high significance and medium effect sizes in differentiation.

**Figure 1:**
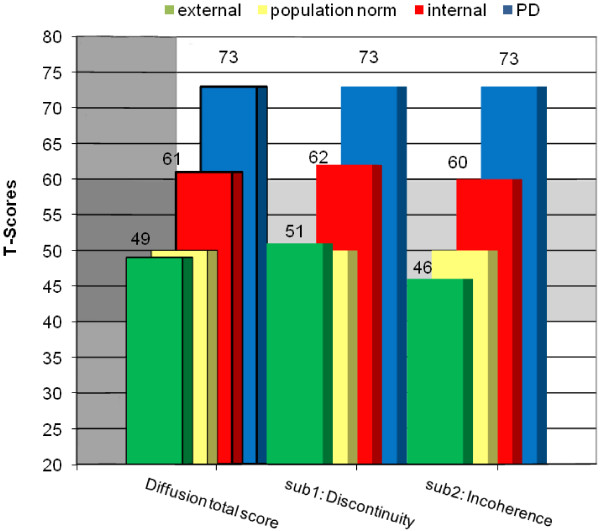
Comparison of T-values in AIDA total and primary scales between the diagnostic groups and the norm population (all T=50).

**Figure 2:**
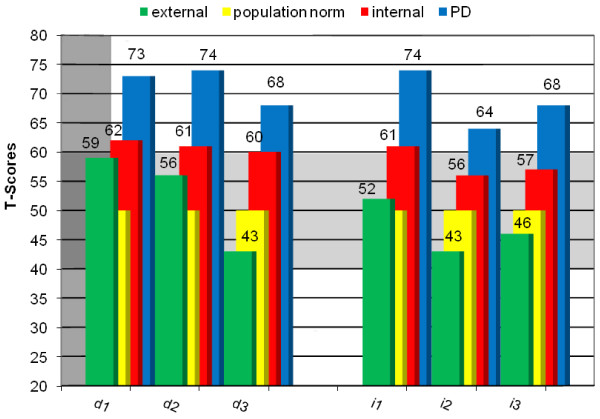
Comparison of T-values in AIDA subscales between the diagnostic groups and the norm population (all T=50).

Figures 1 and 2 are displaying the presented group differences with T-values, thus the meaning of score levels can be interpreted directly. The patients with PD lie clearly above the population norm in their levels of identity diffusion, reflecting a high clinical relevance. The patients with internalizing disorders are slightly above the population norm on total and primary scale level, reflecting an elevated level but below clinical severity, while patients with externalizing disorders do not seem to have systematic differences in their pathology-related identity development compared to a public school sample.

## Discussion

The reformulation of the diagnostic category “Personality Disorders” was one of the highly discussed changes in the revision of DSM-IV to DSM-5. The alternative approach to the diagnosis of personality disorders in Section III of DSM-5 defines a combination of impairments in “self” and “interpersonal” functioning as core criteria of personality disorders. “Self-related personality functioning” is composed of the two constructs “Self-direction” and “Identity”. As indicated by placing the new approach in section III of the new manual further research is recommended to unify the different conceptualizations of personality disorders. To perform this research, valid and reliable tools to assess the core constructs of PD are urgently needed.

The new self-report inventory AIDA assesses pathology-related identity development in adolescence with good reliability and validity [16]. We investigated the power of the inventory to differentiate between adolescents with different psychiatric disorders in respect to normal and disturbed identity development.

In line with our assumptions, the results clearly indicated a high discriminative power of *AIDA* concerning different psychiatric groups, each assigned theoretically with different levels of clinically relevant identity diffusion. The patients with PD, mostly borderline or other B-type, scored not only remarkably higher than the healthy norm population but also higher than the other patient groups with internalizing or externalizing disorders. Moreover, these findings indicate that identity diffusion as it is defined in the AIDA model is a distinguishing mark of PD, not only of psychiatric impairment in general. While patients with PD (Diffusion total score ∅ T= 73) showed highly elevated scores, patients with internalizing disorders, mostly with clinically relevant depression, showed only slightly elevated scores concerning identity diffusion (Diffusion total score ∅ T= 61) and patients with externalizing disorders, mostly diagnosed with ADHD, did not differ from the school population in their identity development at all (Diffusion total score ∅ T= 49).

One of the main aims of *AIDA* is to differentiate between healthy identity integration, current identity crises, and severe identity diffusion. Patients with internalizing disorders scored slightly above the population norm, which may be interpreted as the presence of a current identity crisis. We intended to build homogenous psychiatric groups to also find possible “typical profiles” of identity development and may detect distinct relations between AIDA subscales and type of pathology to help defining the threshold between “crisis” and “diffusion”. But most of the subscales did not differ in their characteristics compared to the primary scales. Thus, further research is needed in this field. Only in the “external” group noticeable differences seemed to occur: patients with externalizing behavior problems had higher levels of “good emotional access to own and others’ feelings” (sub 1.3) and of “autonomy and Ego-strength” (sub 2.2) compared to the healthy controls, while their “stabilizing commitments to interests and goals, subjective selfsameness” (sub 1.1) was nearly as impaired as in the patients of the “internal” group.

It would be comprehensible, however, that patients with externalizing behavior problems (e.g. with conduct disorders) have a relatively consistent self-image (e.g. in terms of a stable criminal identity like “I am a bad guy and feel confident about that.”) and perceive themselves as autonomous (e.g. “I do whatever I want.”), but in our sample only 3 patients with conduct disorder are integrated, thus a separate examination is not possible (see “Limitations” below). With the limited number of patients in the “externalizing disorder” group it is far too early to draw far reaching conclusions from our results. It is essential to enlarge this group with much more patients to be able to differentiate between adolescents with pure ADHD and those with conduct disorder problems.

In general, it is in line with the AIDA-definition of pathology-related identity development that only patients with a personality disorder show elevated scores. The frequently existing artificial overlap in assessing “contradictory behavior” (as part of all descriptions of identity diffusion) and “impulsive behavior” (as part of externalizing behavior), known from a lot of inventories assessing identity-related constructs, is avoided carefully in the questionnaire *AIDA*. Given this, *AIDA* might provide the possibility to differentiate those patients with ADHD from those with emerging antisocial personality disorder.

### Limitations

The criteria for assignment to the three diagnostic groups were strict in order to build homogenous groups. In a classification conference, where we took the results of the diagnostic interviews and clinical experience into account, heterogeneity and comorbidity could be decreased at the cost of a large residual category. This residual category includes 30 of 86 patients which could not be assigned to one of the research groups. Therefore especially the number of patients in the externalizing group was quite low. Furthermore, the group of patients with internalizing problems remains heterogenic. Compared to the other diagnostic groups, the “internal” group shows relatively large standard deviations in their AIDA scores. We can’t exclude that there might be patients in this group who will develop manifest personality disorders in the future. In this study we used the semi-structured diagnostic interview *SCID-II*[39] that has been developed to assess personality disorders in adults. Along with the ongoing revisions of *DSM* and *ICD* it would be very helpful if assessment instruments could be established that are focused on the symptomatology of adolescents with severe impairment of personality functioning.

From a theoretical perspective, it is very useful to know that mean differences in the AIDA scores exist between diagnostic groups, but mean differences do not translate automatically into accurate diagnoses. For diagnostic purposes, we have to consider whether cut-off points regarding identity diffusion and/or crisis might be useful. Once those markers are established, we could determine false positive and false negative rates. Furthermore, when comparing groups, such as adolescents with differing diagnoses, it is important to establish the equivalence of the groups on as many potentially confounding variables as possible. Including more variables (e.g. socio-economic status, level of education, type of parenting received, relationship status of their parents, or arrest records) as well as in-group comparisons or symptom-oriented rearrangements of the sample could lead to new interesting results and show clearly that the differences in the observed identity functioning have more to do with the psychiatric condition than with other variables.

All in all, further research with a bigger sample and even more homogenous groups is needed to highlight distinct profiles and to examine the thresholds between identity crisis and diffusion in detail to develop a more accurate conceptualization of the construct “Identity crisis”. For this aim, longitudinal studies would be of high interest to model the prognostic power of different levels of identity development on subscale level as well as possible changes over time.

## Conclusion

“Identity” is a construct of high interest and is discussed as an essential diagnostic criterion for personality disorders in the new *DSM-5*. For diagnostic purposes, *AIDA* seems to be a useful self-report questionnaire for adolescents from 12 to 18 years to assess pathology-related identity development in terms of this self-related personality function. As patients with personality disorders showed the highest AIDA scores compared to patients with other diagnoses and lied clearly above the population norm in their levels of identity diffusion, remarkable criterion validity can be assumed for this questionnaire and the use of *AIDA* can be recommended for several clinical tasks.

## Competing interests

The authors declare that they have no competing interests.

## Authors’ contributions

EJ and KG were the main writer of the manuscript. KG designed the study and performed the statistical analysis. KS, SS and OP wrote parts of the manuscript. EJ, OP and SS collected the data. All authors read and approved the final manuscript.
